# Effects of tumour budding on adjuvant chemotherapy in colorectal cancer

**DOI:** 10.1093/bjsopen/zrad115

**Published:** 2024-01-08

**Authors:** Hao Xie, Ziwei Zeng, Yujie Hou, Fujin Ye, Tanxing Cai, Yonghua Cai, Li Xiong, Wenxin Li, Zhanzhen Liu, Zhenxing Liang, Shuangling Luo, Xiaobin Zheng, Liang Huang, Huashan Liu, Liang Kang

**Affiliations:** Department of General Surgery (Colorectal Surgery), The Sixth Affiliated Hospital, Sun Yat-sen University, Guangzhou, Guangdong, China; Guangdong Provincial Key Laboratory of Colorectal and Pelvic Floor Diseases, The Sixth Affiliated Hospital, Sun Yat-sen University, Guangzhou, Guangdong, China; Biomedical Innovation Center, The Sixth Affiliated Hospital, Sun Yat-sen University, Guangzhou, Guangdong, China; Guangdong Provincial Key Laboratory of Digestive Cancer Research, The Seventh Affiliated Hospital of Sun Yat-sen University, Shenzhen, Guangdong, China; Department of General Surgery (Colorectal Surgery), The Sixth Affiliated Hospital, Sun Yat-sen University, Guangzhou, Guangdong, China; Guangdong Provincial Key Laboratory of Colorectal and Pelvic Floor Diseases, The Sixth Affiliated Hospital, Sun Yat-sen University, Guangzhou, Guangdong, China; Biomedical Innovation Center, The Sixth Affiliated Hospital, Sun Yat-sen University, Guangzhou, Guangdong, China; Department of General Surgery (Colorectal Surgery), The Sixth Affiliated Hospital, Sun Yat-sen University, Guangzhou, Guangdong, China; Guangdong Provincial Key Laboratory of Colorectal and Pelvic Floor Diseases, The Sixth Affiliated Hospital, Sun Yat-sen University, Guangzhou, Guangdong, China; Biomedical Innovation Center, The Sixth Affiliated Hospital, Sun Yat-sen University, Guangzhou, Guangdong, China; Department of General Surgery (Colorectal Surgery), The Sixth Affiliated Hospital, Sun Yat-sen University, Guangzhou, Guangdong, China; Guangdong Provincial Key Laboratory of Colorectal and Pelvic Floor Diseases, The Sixth Affiliated Hospital, Sun Yat-sen University, Guangzhou, Guangdong, China; Biomedical Innovation Center, The Sixth Affiliated Hospital, Sun Yat-sen University, Guangzhou, Guangdong, China; Department of General Surgery (Colorectal Surgery), The Sixth Affiliated Hospital, Sun Yat-sen University, Guangzhou, Guangdong, China; Guangdong Provincial Key Laboratory of Colorectal and Pelvic Floor Diseases, The Sixth Affiliated Hospital, Sun Yat-sen University, Guangzhou, Guangdong, China; Biomedical Innovation Center, The Sixth Affiliated Hospital, Sun Yat-sen University, Guangzhou, Guangdong, China; Department of General Surgery (Colorectal Surgery), The Sixth Affiliated Hospital, Sun Yat-sen University, Guangzhou, Guangdong, China; Guangdong Provincial Key Laboratory of Colorectal and Pelvic Floor Diseases, The Sixth Affiliated Hospital, Sun Yat-sen University, Guangzhou, Guangdong, China; Biomedical Innovation Center, The Sixth Affiliated Hospital, Sun Yat-sen University, Guangzhou, Guangdong, China; Department of General Surgery (Colorectal Surgery), The Sixth Affiliated Hospital, Sun Yat-sen University, Guangzhou, Guangdong, China; Guangdong Provincial Key Laboratory of Colorectal and Pelvic Floor Diseases, The Sixth Affiliated Hospital, Sun Yat-sen University, Guangzhou, Guangdong, China; Biomedical Innovation Center, The Sixth Affiliated Hospital, Sun Yat-sen University, Guangzhou, Guangdong, China; Department of General Surgery (Colorectal Surgery), The Sixth Affiliated Hospital, Sun Yat-sen University, Guangzhou, Guangdong, China; Guangdong Provincial Key Laboratory of Colorectal and Pelvic Floor Diseases, The Sixth Affiliated Hospital, Sun Yat-sen University, Guangzhou, Guangdong, China; Biomedical Innovation Center, The Sixth Affiliated Hospital, Sun Yat-sen University, Guangzhou, Guangdong, China; Department of General Surgery (Colorectal Surgery), The Sixth Affiliated Hospital, Sun Yat-sen University, Guangzhou, Guangdong, China; Guangdong Provincial Key Laboratory of Colorectal and Pelvic Floor Diseases, The Sixth Affiliated Hospital, Sun Yat-sen University, Guangzhou, Guangdong, China; Biomedical Innovation Center, The Sixth Affiliated Hospital, Sun Yat-sen University, Guangzhou, Guangdong, China; Department of General Surgery (Colorectal Surgery), The Sixth Affiliated Hospital, Sun Yat-sen University, Guangzhou, Guangdong, China; Guangdong Provincial Key Laboratory of Colorectal and Pelvic Floor Diseases, The Sixth Affiliated Hospital, Sun Yat-sen University, Guangzhou, Guangdong, China; Biomedical Innovation Center, The Sixth Affiliated Hospital, Sun Yat-sen University, Guangzhou, Guangdong, China; Department of General Surgery (Colorectal Surgery), The Sixth Affiliated Hospital, Sun Yat-sen University, Guangzhou, Guangdong, China; Guangdong Provincial Key Laboratory of Colorectal and Pelvic Floor Diseases, The Sixth Affiliated Hospital, Sun Yat-sen University, Guangzhou, Guangdong, China; Biomedical Innovation Center, The Sixth Affiliated Hospital, Sun Yat-sen University, Guangzhou, Guangdong, China; Department of General Surgery (Colorectal Surgery), The Sixth Affiliated Hospital, Sun Yat-sen University, Guangzhou, Guangdong, China; Guangdong Provincial Key Laboratory of Colorectal and Pelvic Floor Diseases, The Sixth Affiliated Hospital, Sun Yat-sen University, Guangzhou, Guangdong, China; Biomedical Innovation Center, The Sixth Affiliated Hospital, Sun Yat-sen University, Guangzhou, Guangdong, China; Department of General Surgery (Colorectal Surgery), The Sixth Affiliated Hospital, Sun Yat-sen University, Guangzhou, Guangdong, China; Guangdong Provincial Key Laboratory of Colorectal and Pelvic Floor Diseases, The Sixth Affiliated Hospital, Sun Yat-sen University, Guangzhou, Guangdong, China; Biomedical Innovation Center, The Sixth Affiliated Hospital, Sun Yat-sen University, Guangzhou, Guangdong, China; Department of General Surgery (Colorectal Surgery), The Sixth Affiliated Hospital, Sun Yat-sen University, Guangzhou, Guangdong, China; Guangdong Provincial Key Laboratory of Colorectal and Pelvic Floor Diseases, The Sixth Affiliated Hospital, Sun Yat-sen University, Guangzhou, Guangdong, China; Biomedical Innovation Center, The Sixth Affiliated Hospital, Sun Yat-sen University, Guangzhou, Guangdong, China; Guangdong Provincial Key Laboratory of Digestive Cancer Research, The Seventh Affiliated Hospital of Sun Yat-sen University, Shenzhen, Guangdong, China; Department of General Surgery (Colorectal Surgery), The Sixth Affiliated Hospital, Sun Yat-sen University, Guangzhou, Guangdong, China; Guangdong Provincial Key Laboratory of Colorectal and Pelvic Floor Diseases, The Sixth Affiliated Hospital, Sun Yat-sen University, Guangzhou, Guangdong, China; Biomedical Innovation Center, The Sixth Affiliated Hospital, Sun Yat-sen University, Guangzhou, Guangdong, China

## Abstract

**Background:**

High tumour budding has been indicated as a risk factor of poor survival in colorectal cancer. This study aimed to investigate the impact of tumour budding grades and the use of adjuvant chemotherapy on prognosis in patients with colorectal cancer.

**Methods:**

This study included consecutive colorectal cancer patients who underwent radical surgery for primary colorectal adenocarcinoma at The Sixth Hospital of Sun Yat-sen University between 2009 and 2019. Tumour budding was assessed based on the recommendations of the International Tumor Budding Consensus Conference using haematoxylin and eosin (H&E)-stained slides with tumour samples. The primary outcome of interest was to correlate tumour budding with disease-free survival and overall survival; the secondary outcome was investigation of the impact of adjuvant therapy on different tumour budding grades. In addition, a subgroup analysis was performed for the effects of lymphocytic infiltration on adjuvant chemotherapy in patients with Bd3.

**Results:**

Of 709 eligible patients, 412 with colorectal cancer were included. According to the International Tumor Budding Consensus Conference, 210 (50.9 per cent), 127 (30.8 per cent) and 75 (18.2 per cent) were classified as low budding (Bd1), intermediate budding (Bd2) and high budding (Bd3) respectively. Patients with Bd1, Bd2 and Bd3 had 5-year disease-free survival rates of 82.9 per cent, 70.1 per cent and 49.3 per cent respectively, and 5-year overall survival rates of 90 per cent, 79.5 per cent and 62.7 per cent respectively (*P* <0.001). Adjuvant chemotherapy yielded a significant survival benefit in patients with Bd3 (5-year disease-free survival, 65 per cent *versus* 31.4 per cent, *P* <0.001; 5-year overall survival, 84.4 per cent *versus* 63.1 per cent, *P* <0.001), but not in those with Bd1 or Bd2. In patients with Bd3, the benefit of adjuvant chemotherapy was maintained in those with low, but not high lymphocytic infiltration.

**Conclusion:**

High grade of tumour budding was strongly correlated with poorer survival outcomes in colorectal cancer. Patients with Bd3 benefited from adjuvant chemotherapy, with the exclusion of patients with high lymphocytic infiltration.

## Teaser

In this study, we demonstrated that patients with Bd3 might benefit from adjuvant chemotherapy. The benefit of chemotherapy was maintained in patients with Bd3 in the low lymphocytic infiltration group, while there was no tendency for chemotherapy to have a positive effect in the high lymphocytic infiltration group.

## Introduction

Patients with colorectal cancer (CRC) often receive postoperative chemotherapy to improve their prognosis^1–4^. According to National Comprehensive Cancer Network (NCCN) guidelines, adjuvant chemotherapy is recommended for patients with stage III CRC^5,6^. However, a recent real-world study involving more than 200 000 patients found that only 20 per cent of stage III CRC patients actually benefit from adjuvant chemotherapy^7^. Nearly 80 per cent of patients may be subjected to the unnecessary hazards and toxicity associated with multiagent chemotherapy^8^. On the other hand, a significant proportion of stage II CRC patients treated with surgery alone report a 5-year survival rate of approximately 68 per cent, suggesting that additional therapy might have the potential to improve their prognosis^9^. Therefore, there is an unmet clinical need to identify CRC patients who could benefit from adjuvant treatment.

Tumour budding (TB) was first described by Imai in the 1950s^10^ and is defined as the presence of single cancer cells or clusters of up to four cells at the invasive tumour front^11^. It has been found to be strongly correlated with higher tumour grade, the presence of lymph node metastases, perineural invasion and immune escape^12,13^. Five independent studies demonstrated that patients with low-grade tumour budding (Bd1) had a 5-year disease-specific survival (DSS) of 89–98 per cent, while patients with intermediate-grade (Bd2) or high-grade tumour budding (Bd3) have a significantly worse 5-year DSS of 52–80 per cent^14–18^. These results suggested that adjuvant chemotherapy may be beneficial for patients with a higher grade of TB to improve their prognosis. In addition, previous studies showed a negative correlation between the number of TB and lymphocytic infiltration (LI), and the effectiveness of chemotherapy may also depend, in part, on LI^19,20^. Therefore, this study aimed to investigate the impact of TB and adjuvant chemotherapy on survival in CRC patients who underwent radical surgery.

## Methods

### Study design and patients

This study retrospectively included patients who underwent surgery for primary colorectal adenocarcinoma at The Sixth Hospital of Sun Yat-sen University between January 2009 and December 2019. Patients with neoadjuvant treatment, recurrent disease, stage IV, hereditary nonpolyposis CRC, palliative surgery, local resection or insufficient follow-up data were excluded. The Institutional Review Board of Sun Yat-sen University approved this study, which was registered at clinicaltrials.gov as NCT05610592.

### TB assessment

The assessment of TB was performed by two independent pathologists who were blinded to the patients’ clinical information using haematoxylin and eosin (H&E)-stained slides, in accordance with the guidelines established by the International Tumor Budding Consensus Conference (ITBCC)^21^. The normalized number of buds was used to assign a TB grade, with Bd1 for 0 to 4 buds, Bd2 for 5 to 9 buds, and Bd3 for ≥10 buds (*Supplementary Materials*, *Fig. S1*). All pathology specimens were reviewed for TB assessment in all patients for the purpose of this study and categorized as Bd3 (high grade) and Bd1 and Bd2 (low grade). In CRC, multiple tumour blocks are typically available for TB counting, and section thickness can vary within the same tumour block. To assess the precision of TB counting within the tumor, TB was evaluated at 13 levels distributed from section 1 to section 100 (adjacent slides of section levels 1, 3, 5, 7, 9, 20, 30, 40, 50, 60, 70, 80 and 99) from each tissue block^22^. TB was also evaluated in six tissue blocks from the same patient to investigate intertumoral correlation.

### LI evaluation

The density of CD3^+^ and CD8^+^ cells in both the centre of tumour (CT) and invasive margin (IM) of the tumour on each immunohistochemical slide was used to calculate the CD3^+^ and CD8^+^ cell density as the number per mm^2^ (*Supplementary Materials*, *Fig. S2*). The LI assessment was performed based on the densities of CD3^+^ and CD8^+^ T cells in both the CT and IM regions. IL evaluations were conducted in all patients for the purpose of this study. To determine the cut-off that best separated patient groups in terms of their disease-free survival, the ‘minimum *P* value’ approach was used (*Supplementary Materials*, *Table S1*), and all scores were calculated to evaluate LI^23^. A score of 1 was assigned for a high value of each index, while a score of 0 was assigned for a low value. A score of 4 represented a high density of both lymphocyte types in both regions, while a score of 0 corresponded to a low density of CD3^+^ and CD8^+^ T cells in both CT and IM. Patients with a score >2 were defined as having high LI, while those with a score ≤2 were defined as having low LI^24,25^.

### Treatment and outcomes

All treatments were administered based on individual patient considerations after multidisciplinary team discussions and were guided by the guidelines recommended by the National Health Commission of the People's Republic of China. All patients included in this study underwent curative surgery for CRC, and postoperative therapy was routinely recommended for patients with pathologic T4, N0 and T1–4, N+ disease. The postoperative chemotherapy regimens included CAPEOX (oxaliplatin and capecitabine) and FOLFOX (oxaliplatin, 5-Fu, and leucovorin (LV)). The study assessed two follow-up outcomes: disease-free survival (DFS) and overall survival (OS). DFS was defined as the proportion of patients who survived without local or distant cancer recurrence at the time of review, while OS was defined as the proportion of patients who were alive at the time of censoring. The follow-up interval for all patients was from the date of surgery to the last censoring date (at least 5 years). The demographic (age and sex), pathologic (tumour location, TNM stage, tumour deposits, lymphovascular invasion and perineural invasion) and follow-up data were collected from electronic medical records.

The primary outcome of interest of this research was to correlate TB with survival; the secondary outcome was investigation of the impact of adjuvant therapy for different TB grades after balancing for confounding factors. In addition, a subgroup analysis was performed for the effects of LI on adjuvant chemotherapy in patients with Bd3.

### Statistical analysis

Statistical analyses were conducted using SPSS (version 25.0, IBM). Normally distributed data were reported as mean(s.d.) and analysed using the Student's *t* test, while non-normally distributed data were presented as median (interquartile range) and analysed using appropriate non-parametric tests, such as the Mann–Whitney test or Kruskal–Wallis test. The Maximally Selected Log-rank Statistic was used to calculate the cut-off of the densities of CD3^+^ and CD8^+^ T cells in both the CT and IM regions to separate patient groups in terms of their DFS. The interobserver agreement was calculated using Pearson’s r. To assess the relationship between lymphocytes and TB, Spearman correlation coefficients (r) were applied on continuous data. All r = 0 values indicated no association between features, r >0 indicated a positive association and r <0 indicated a negative association. The relationship between lymphocyte distribution patterns and the grade of TB was assessed using the χ^2^ test. For follow-up analyses, the follow-up interval was limited to 5 years, and patients without events within 5 years were right-censored. Prognosis in different groups was estimated using the Kaplan–Meier method, and statistical comparisons were performed using the log-rank test for the following variables: TB grades and treatment. Subgroup analyses for the association between TB and survival outcomes were conducted to balance confounding factors (TNM stage, tumour location, mismatch repair (MMR) and LI status). Similar analyses were also conducted for the association between adjuvant therapy and survival outcomes. The Cox proportional hazard regression was used to model the prognostic relationships between TB and outcomes, and the Cox model interaction tests were performed to examine the predictive effects of TB with treatment. Hazard ratios (HRs) and 95 per cent confidence intervals (c.i.) were calculated, and *P* values less than 0.05 were considered statistically significant.

## Results

### Characteristics of patients

Of 709 eligible patients, a total of 412 patients with stage I–III CRC were included in this study (*Supplementary Materials*, *Fig. S3*), of whom 244 (59 per cent) were male, and the median age was 58 years (interquartile range (i.q.r.): 48–68). Increasing grades of TB were associated with higher proportions of T3–4, positive lymph nodes, tumour deposits, lymphovascular invasion, perineural invasion and mismatch repair-proficient (pMMR) status (*P* = 0.008, *P* <0.001, *P* <0.001, *P* <0.001, *P* = 0.005 and *P* = 0.008 respectively) (*Table 1*). Among the patients, 239 (58 per cent) underwent surgery alone, and 173 (42 per cent) received surgery and adjuvant chemotherapy. The median postoperative follow-up time was 58 months (range, 1–144 months). At the last follow-up, 75 (18 per cent) patients had died, and recurrence had occurred in 74 patients (18 per cent), including pulmonary metastases in 17 patients (23 per cent), liver metastases in 12 patients (16 per cent), non-regional lymph node metastases in 8 patients (11 per cent), metastases involving two or more organs in 18 patients (24 per cent), and local recurrence in 19 patients (26 per cent).

**Table 1:** Clinicopathologic features and their correlations with tumour budding grades

	Total	Bd1	Bd2	Bd3	*P*
	(*n* = 412)	(*n* = 210 (50.9))	(*n* = 127 (30.8))	(*n* = 75 (18.2))	
Age (years), mean(s.d.)	58(10)	58(10)	58(9)	60(12)	0.724
**Sex**					
Male	244 (59.2)	124 (59)	75 (59.1)	45 (60)	0.989
**Tumour location**					
Colon	185 (44.9)	106 (50.5)	50 (39.4)	29 (36.7)	0.068
Rectum	227 (55.1)	104 (49.5)	77 (60.6)	46 (61.3)	
**pT Stage**					
T1–T2	82 (19.9)	53 (25.2)	22 (17.3)	7 (9.3)	**0.008**
T3–T4	330 (80.1)	157 (74.8)	105 (82.7)	68 (90.7)	
**pN Stage**					
N0	252 (61.2)	156 (74.3)	71 (55.9)	25 (33.3)	**<0**.**001**
N1–2	160 (38.8)	54 (25.7)	56 (44.1)	50 (66.7)	
**Tumour deposits**					
Negative	352 (85.4)	171 (91)	111 (85)	47 (70.7)	**<0**.**001**
Positive	60 (14.6)	19 (9)	19 (15)	22 (29.3)	
**Lymphovascular invasion**					
Negative	366 (88.8)	196 (93.3)	115 (90.6)	55 (73.3)	**<0**.**001**
Positive	46 (11.2)	14 (6.7)	12 (9.4)	20 (26.7)	
**Perineural invasion**					
Negative	363 (88.1)	192 (91.4)	113 (89)	58 (77.3)	**0**.**005**
Positive	49 (11.9)	18 (8.6)	14 (11)	17 (22.7)	
**LI**					
Low	193 (46.8)	82 (39)	65 (51.2)	46 (61.3)	**0**.**002**
High	219 (53.2)	128 (61)	62 (48.8)	29 (38.7)	
**MMR status**					
dMMR	223 (55)	128 (65.7)	64 (52)	31 (46.7)	**0**.**008**
pMMR	189 (45)	82 (34.3)	63 (48)	44 (53.3)	

Values in parentheses are percentages unless indicated otherwise; bolded values: significant *P* values. Bd1, low budding; Bd2, intermediate budding; Bd3, high budding; LI, lymphocytic infiltration; pT stage, pathologic T stage; pN stage, pathologic N stage; MMR, mismatch repair; dMMR, mismatch repair-deficient; pMMR, mismatch repair-proficient.

### TB and survival

**Fig. 1:**
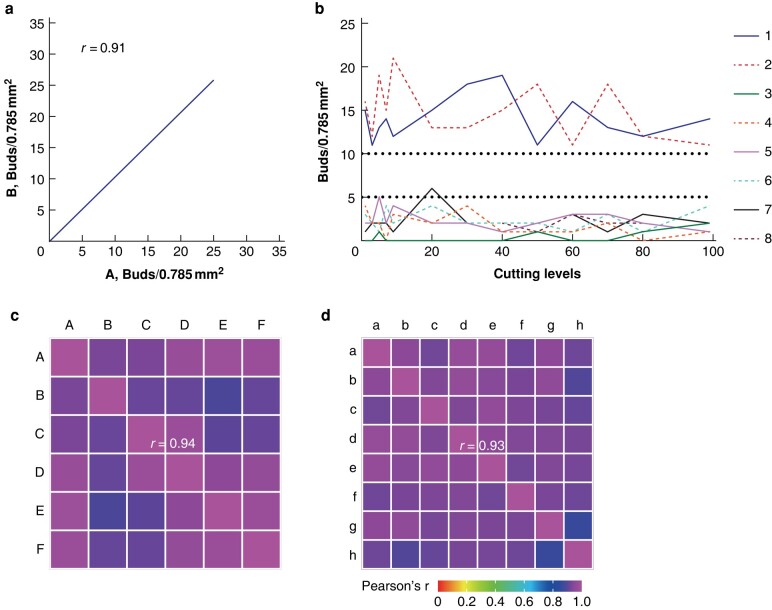
**Robustness of tumour budding counting** **a** Example of 2 by 2 correlation of the tumour bud counts between two pathologists. **b** A total of 13 levels (1, 3, 5, 7, 9, 20, 30, 40, 50, 60, 70, 80, 99) of cutting from 100 adjacent cuts of each tumour block (*n* = 8) were investigated for tumour buds. **c** Correlation matrices illustrating the precision of the tumour bud counts in 6 tumour blocks of colorectal cancer. The mean of all 2 × 2 correlations between the 6 tumour blocks is r = 0.94. **d** Correlation matrices illustrating the precision of the tumour bud counts in 13 levels (1, 9, 20, 30, 40, 50, 60, 70, 80) of cutting from 100 adjacent cuts of each tumour block. The mean of all 2 × 2 correlations between the 6 tumour blocks is r = 0.93.

**Fig. 2:**
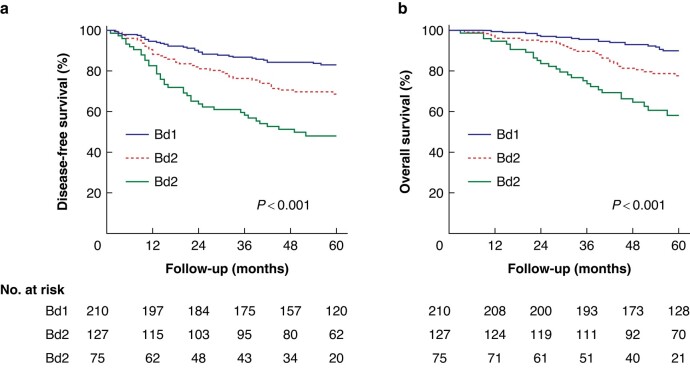
**Survival outcomes according to TB in all patients** **a** Disease-free survival and **b** overall survival according to TB in all patients. TB, tumour budding.

The level of interobserver agreement in tumour bud counting was evaluated using H&E-stained slides (Pearson r = 0.92), indicating strong agreement among observers (*Fig. 1a*). Minimal variation in tumour buds across sections was observed in all tumours, with only one case exhibiting a shift from one TB category to another in blocks where tumour bud counts were very close to the intersection between two categories (*Fig. 1b*). The correlation between intratumoral and intertumoral tissue blocks was depicted in the correlation matrices using Pearson’s correlation coefficient (r) (*Fig. 1c*, *d*). Patients with Bd1, Bd2 and Bd3 had 5-year DFS rates of 82.9 per cent, 70.1 per cent and 49.3 per cent respectively, and 5-year OS rates of 90 per cent, 79.5 per cent and 62.7 per cent respectively (*P* <0.001) (*Fig. 2*). After stratifying by TNM stage (I, II or III), anatomic location (colon or rectum), MMR status (mismatch repair-deficient [dMMR] or pMMR) and LI (LI-Hi or LI-Lo), a higher grade of TB was still significantly associated with worse survival outcomes (*Supplementary Materials*, *Fig. S4–S8*). Univariable and multivariable analyses confirmed that N1–2 stage, perineural invasion and higher TB predicted worse survival outcomes (*Supplementary Materials*, *Table S2*). Together, TB was a robust independent prognostic marker for patients with CRC.

### Association between TB and adjuvant chemotherapy in CRC patients

**Fig. 3:**
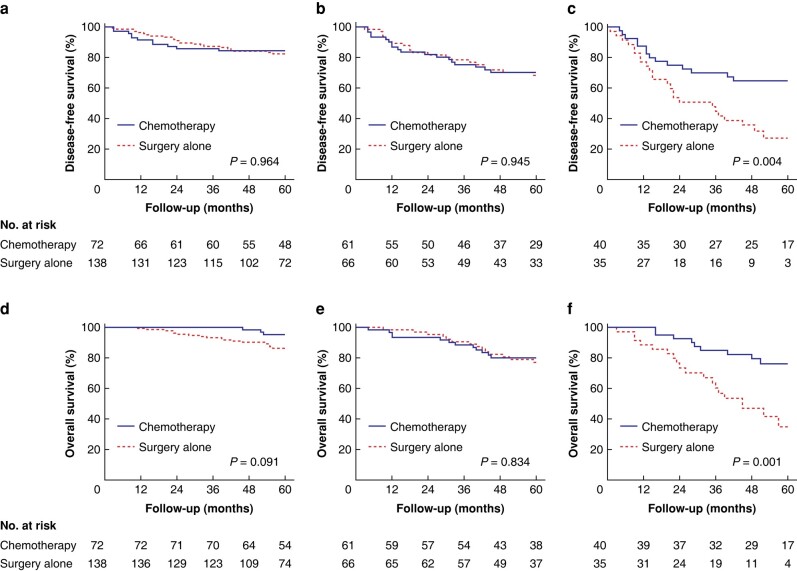
**Survival outcomes according to treatment in patients with Bd1, Bd2 and Bd3** Disease-free survival according to treatment in **a** Bd1, **b** Bd2, and **c** Bd3 groups in all patients. Overall survival according to treatment in **d** Bd1, **e** Bd2, and **f** Bd3 groups in all patients. Bd1, low budding; Bd2, intermediate budding; Bd3, high budding.

Given that patients with a higher grade of TB had worse survival, the association between TB and efficacy of adjuvant chemotherapy was further investigated. To this end, patients were divided into three groups based on the three-tiered system. Results showed that 210 (50.9 per cent), 127 (30.8 per cent) and 75 (18.2 per cent) patients were classified as Bd1, Bd2 and Bd3 respectively. With the exceptions of age, T stage, N stage, tumour deposits and MMR status, the characteristics across patients with Bd1, Bd2 and Bd3 were balanced in the groups with chemotherapy and surgery alone (*Supplementary Materials*, *Table S3*). The survival outcomes between patients who received adjuvant chemotherapy and those who did not were then compared and stratified by the degree of TB. The results demonstrated that adjuvant chemotherapy correlated with a significant survival benefit for patients with Bd3 (5-year DFS, 65 per cent *versus* 31.4 per cent; *P* <0.001), but not for patients with Bd1 (81.9 per cent *versus* 83.3 per cent; *P* = 0.796) or Bd2 (70.5 per cent *versus* 65.7 per cent; *P* = 0.953) (*Fig. 3a–c*). Moreover, OS analyses indicated that the survival rate was significantly higher in the chemotherapy group than in the surgery alone group for patients with Bd3 (5-year OS, 84.4 per cent *versus* 63.1 per cent; *P* <0.001). However, there were no differences in 5-year OS between the chemotherapy and surgery alone groups for patients with Bd1 (94.4 per cent *versus* 87.7 per cent; *P* = 0.09) or Bd2 (80.3 per cent *versus* 78.8 per cent; *P* = 0.838) (*Fig. 3e–g*). In addition, the benefit of adjuvant chemotherapy in patients with Bd3 was observed regardless of MMR status, tumour location and TNM stage (*Supplementary Materials*, *Fig. S9–S11*).

**Fig. 4:**
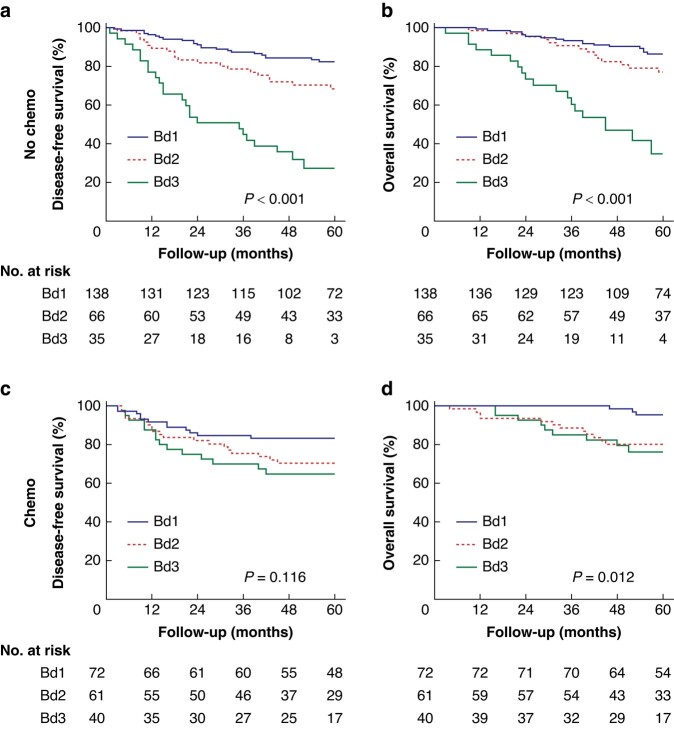
**Survival outcomes according to tumour budding in patients with chemotherapy or not** **a** Disease-free survival and **b** overall survival according to tumour budding in patients who underwent surgery alone. **c** Disease-free survival and **d** overall survival according to tumour budding in patients who received adjuvant chemotherapy.

To further describe the association between TB and adjuvant therapy, survival outcomes differing by TB grades in patients who received or did not receive adjuvant chemotherapy were compared. These results showed a significant association between TB and survival outcomes (*Fig. 4a*, *b*), but this association was not significant in patients who underwent adjuvant chemotherapy (*P* = 0.116) (*Fig. 4c*, *d*). In the multivariable Cox models, the interaction between TB and adjuvant chemotherapy remained significant for DFS (*P* = 0.018) even after adjusting for all clinical and histopathological features (*Table 2*).

**Table 2:** Multivariate Cox analysis with interaction between tumour budding grades and chemotherapy on DFS and OS

Disease-free survival			Overall survival	
	Multivariable HR (95% c.i.)	*P*	Multivariable HR (95% c.i.)	*P*
**pT stage**				
T1–T2	1	**0**.**031**	1	0.054
T3–T4	2.1 (1.07,4.11)		2.33 (0.98,5.51)	
**pN stage**				
N0	1	**0**.**003**	1	**0**.**019**
N1–N2	2.06 (1.28,3.32)		2.02 (1.12,3.64)	
**Tumour deposits**				
Negative	1	0.119	1	0.457
Positive	1.48 (0.91,2.42)		1.26 (0.68,2.31)	
**Lymphovascular invasion**				
Negative	1	0.297	1	0.282
Positive	1.32 (0.78,2.26)		1.42 (0.74,2.72)	
**Perineural invasion**				
Negative	1	**0**.**004**	1	**0**.**001**
Positive	1.99 (1.25,3.16)		2.47 (1.42,4.68)	
**LI**				
Low	1	0.067	1	0.874
High	0.69 (0.47,1.02)		0.96 (0.6,1.54)	
**TBG**				
Low	1	**<0**.**001**	1	**<0**.**001**
High	2.84 (1.86,4.86)		3.46 (1.83,6.51)	
**Chemo**				
No	1	0.121	1	**0**.**011**
Yes	0.66 (0.41,1.09)		0.43 (0.22,0.81)	
TBG:Chemo	0.37 (0.16,0.84)	**0**.**018**	0.39 (0.13,1.11)	0.077

DFS, disease-free survival; OS, overall survival; pT stage, pathologic T stage; pN stage, pathologic N stage; LI, lymphocytic infiltration; Chemo, chemotherapy; TBG, tumour budding grades; TBG:Chemo, interaction term between tumour budding grades and chemotherapy; HR, hazard ratio; bolded values, significant *P* values.

### Effects of LI on adjuvant chemotherapy in patients with Bd3

**Fig. 5:**
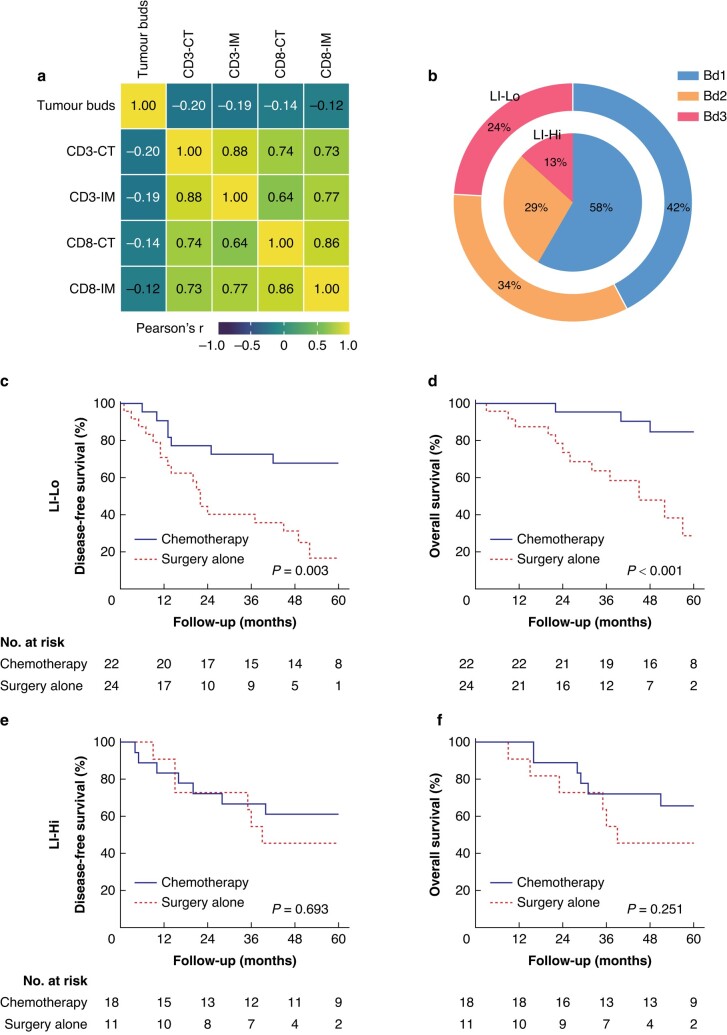
**Effects of LI on chemotherapy in patients with Bd3** **a** Spearman correlation matrix for lymphocyte densities and TB numbers. Spearman correlation coefficient is shown for all relationships. **b** Ring charts illustrating the proportion of Bd1, Bd2, and Bd3 in high LI and low LI group. **c** Disease-free survival and **d** overall survival according to treatment in Bd3 group in patients with low LI. **e** Disease-free survival and **f** overall survival according to treatment in Bd3 group in patients with High LI. TB, tumour budding; LI, lymphocytic infiltration; Bd1, low budding; Bd2, intermediate budding; Bd3, high budding.

Since a high grade of LI was associated with better survival outcome (*Supplementary Materials*, *Fig. S12*, *S13*), the effects of LI on chemotherapy in patients with Bd3 were investigated further. Firstly, there was a negative correlation between the number of tumour buds (TB) and all lymphocyte densities, regardless of their distribution pattern (intratumoral or peritumoral) or lymphocyte subpopulations (CD3^+^ or CD8^+^) (*Fig. 5a*, *Supplementary Materials, Fig. S14*). Furthermore, high LI was significantly correlated with lower TB (*Fig. 5b*). Additionally, the benefit of chemotherapy was maintained in patients with Bd3 in the low LI group (*P* = 0.003), while in the high LI group, there was no significant difference in survival outcomes between patients who received chemotherapy and those who did not (*P* = 0.251) (*Fig. 5c*, *d*).

## Discussion

The selection of appropriate patients for adjuvant treatment in CRC remains an unmet clinical need. The limited efficacy of adjuvant chemotherapy in CRC patients emphasizes the need for effective markers to identify those who would benefit from treatment^26^. This study demonstrated a significant association between TB and tumour recurrence and mortality. Furthermore, patients with Bd3 who received adjuvant chemotherapy exhibited better survival outcomes than those who underwent surgery alone.

TB is a highly invasive tumour subpopulation that can evade the immune response^27,28^. As an emerging prognostic biomarker in CRC^11,29,30^, it has been strongly correlated with higher tumour grade, the presence of lymph node metastases and perineural invasion^12,13^. The prognostic value of TB has been validated in five independent studies, involving a total of 1978 patients with stage II CRC^14–18^. Patients with low-grade tumour budding (Bd1) had a 5-year DSS of 89–98 per cent, while patients with intermediate-grade (Bd2) or high-grade TB (Bd3) had a significantly worse 5-year DSS of 52–80 per cent. In line with previous studies, a higher grade of TB was significantly associated with worse survival outcomes, even when stratified by TNM stage (I, II or III), anatomic location (colon or rectum), MMR status (dMMR or pMMR) and LI (LI-Hi or LI-Lo).

The National Comprehensive Cancer Network, European Society for Medical Oncology and American Society of Clinical Oncology have issued guidelines recommending the use of high-risk clinicopathologic features, such as poorly differentiated/undifferentiated histology, lymphatic/vascular invasion, bowel obstruction, < 12 examined lymph nodes, perineural invasion, localized perforation, narrow or indeterminate positive margins, or TB, to select patients for adjuvant chemotherapy^1,5,29^. However, previous studies have not demonstrated the predictive utility of these high-risk features for the benefit of adjuvant chemotherapy, nor have there been any studies linking specific features to the choice of chemotherapy. Our study evaluated the predictive value of TB in response to chemotherapy in patients with CRC by comparing the survival outcomes between groups with chemotherapy and surgery alone groups. The findings presented here suggest that adjuvant chemotherapy is beneficial for patients with Bd3 but not for those with Bd1 or Bd2.

The effectiveness of chemotherapy also depends, in part, on the density of tumour-infiltrating T cells. A previous study reported a significant association between chemotherapy and survival in the high-immunoscore group for both low-risk and high-risk patients. However, in their study, none of the few patients with the highest immunoscore relapsed even when they were not treated with chemotherapy^20^. In the present study, there was no tendency for chemotherapy to have a positive effect in patients with Bd3 in the high LI group. These results suggest that the use of chemotherapy in patients with a high grade of LI could be discussed.

There are limitations to this study that should be considered. First, it is a retrospective study conducted at a single centre, which may limit the generalizability of our conclusions. Larger, prospective clinical studies are needed to validate these findings and draw more reliable conclusions. Second, patients’ backgrounds varied between the chemotherapy and surgery alone groups. Patients who were physically unsuitable for adjuvant chemotherapy due to age, complications or other reasons were assigned to the surgery alone group. However, the interaction between TB and adjuvant chemotherapy remained significant even after adjusting for all clinical and histopathological features in the multivariable Cox models. Finally, the interaction between TB and FOXP3^+^ T cells and CD68^+^ macrophages, which are also frequently detected immune cells in TB regions^30^, was not considered. Future studies may want to consider a combination of several biomarkers of the TB microenvironment to improve classification.

## Supplementary Material

zrad115_Supplementary_Data

## Data Availability

The data derived from the electronic medical record system in The Sixth Affiliated Hospital, Sun Yat-sen University. Due to the sensitive nature of the data in this study, raw data, information and data of the patients will remain confidential and not be shared.
