# Supplementary material for: Effects of tumour budding on adjuvant chemotherapy in colorectal cancer
Source: BJS Open. 2024 Jan 8;8(1):zrad115. doi: 10.1093/bjsopen/zrad115 (PMC10773627; doi:10.1093/bjsopen/zrad115)
Supplement: zrad115_Supplementary_Data [file zrad115_supplementary_data.docx]

**Title: Effects of Tumor Budding on Adjuvant Chemotherapy in Colorectal Cancer**

Authors: Hao Xie^1, 2, 3, 4*^, Ziwei Zeng^1, 2, 3*^, Yujie Hou^1, 2, 3*^, Fujin Ye^1, 2, 3^, Tanxing Cai^1, 2, 3^, Yonghua Cai^1, 2, 3^, Xiong Li^1, 2, 3^, Wenxin Li^1, 2, 3^, Zhanzhen Liu^1, 2, 3^, Zhenxing Liang^1, 2, 3^, Shuangling Luo^1, 2, 3^, Xiaobin Zheng^1, 2, 3^, Liang Huang^1, 2, 3#^, Huashan Liu^1, 2, 3, 4#^, Liang Kang^1, 2, 3#^

^1^Department of General Surgery (Colorectal Surgery), The Sixth Affiliated Hospital, Sun Yat-sen University, Guangzhou, Guangdong, China.

^2^Guangdong Provincial Key Laboratory of Colorectal and Pelvic Floor Diseases, The Sixth Affiliated Hospital, Sun Yat-sen University, Guangzhou, Guangdong, China.

^3^Biomedical Innovation Center, The Sixth Affiliated Hospital, Sun Yat-sen University, Guangzhou, Guangdong, China.

^4^Guangdong Provincial Key Laboratory of Digestive Cancer Research, the Seventh Affiliated Hospital of Sun Yat-sen University, Shenzhen, Guangdong, China.

**Corresponding author:**

**Liang Kang, MD, PhD; Department of General Surgery (Colorectal Surgery), The Sixth Affiliated Hospital, Sun Yat-sen University, Guangzhou, Guangdong, China; Email: kangl@mail.sysu.edu.cn; ORCID ID: 0000-0001-7062-8280.**

**Huashan Liu, MD, PhD; Department of General Surgery (Colorectal Surgery), The Sixth Affiliated Hospital, Sun Yat-sen University, Guangzhou, Guangdong, China; Email:** **liuhshan@mail2.sysu.edu.cn.**

**Liang Huang, MD, PhD; Department of General Surgery (Colorectal Surgery), The Sixth Affiliated Hospital, Sun Yat-sen University, Guangzhou, Guangdong, China; Email: huangl75@mail.sysu.edu.cn.**

**Supplementary Materials - Index**

| **Supplementary Figures and Tables** |  |
| --- | --- |
| Figure S1 | *pag. 3* |
| Figure S2  Figure S3  Figure S4  Figure S5  Figure S6  Figure S7  Figure S8  Figure S9  Figure S10  Figure S11  Figure S12  Figure S13  Figure S14  Table S1  Table S2  Table S2 | *pag. 4*  *pag. 5*  *pag. 6*  *pag. 7*  *pag. 8*  *pag. 9*  *pag. 10*  *pag. 11*  *pag. 12*  *pag. 13*  *pag. 14*  *pag. 15*  *pag. 16*  *pag. 17*  *pag. 18*  *pag. 19* |

**Supplementary Figures and Tables**


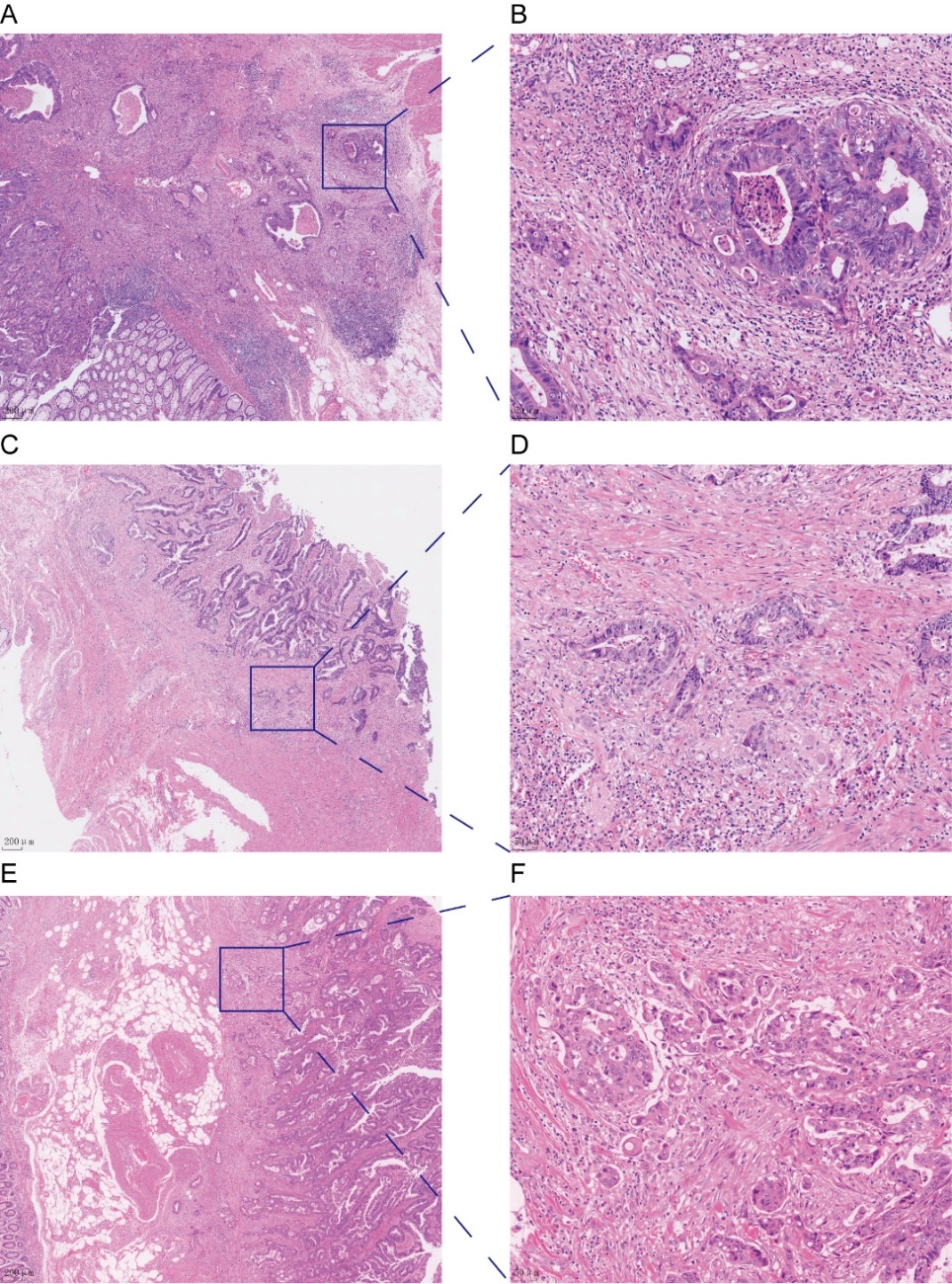


**Figure S1.** Tumor budding images (hematoxylin and eosin)

Panels A and B show representative examples of tumor budding grade 1 seen at low magnification and higher power (40 **×** and 200 **×**).

Panels C and D show representative examples of tumor budding grade 2 seen at low magnification and higher power (40 **×** and 200 **×**).

Panels E and F show representative examples of tumor budding grade 3 seen at low magnification and higher power (40 **×** and 200 **×**).

**
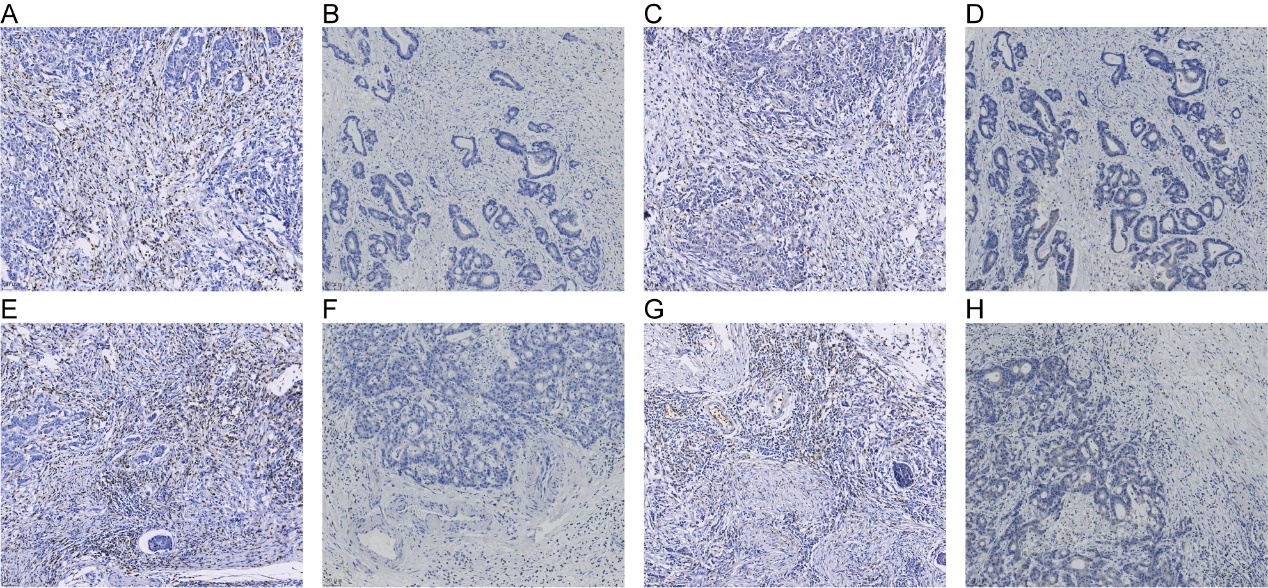
**

**Figure S2.** Representative immunohistochemical images of CD3+ and CD8+ T cells in the center tumor (CT) and in the invasive margin (IM) of the colorectal cancer

A-B, Representative images of high-density and low-density CD3+ cells in the center tumor of the colorectal cancer;

C-D, Representative images of high-density and low-density CD3+ cells in the invasive margin of tumor;

E-F, Representative images of high-density and low-density CD8+ cells in the center tumor of the colorectal cancer;

G-H, Representative images of high-density and low-density CD8+ cells in the invasive margin.


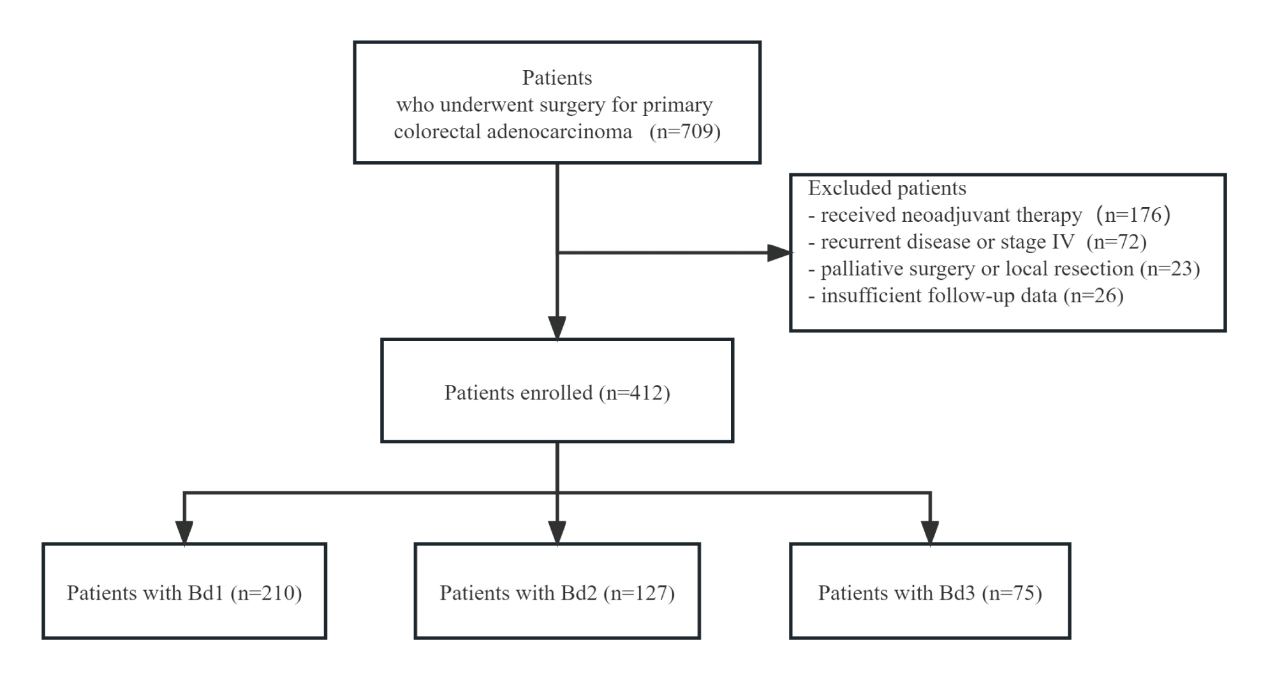


**Figure S3.** Flow-chart of included and excluded patients

**
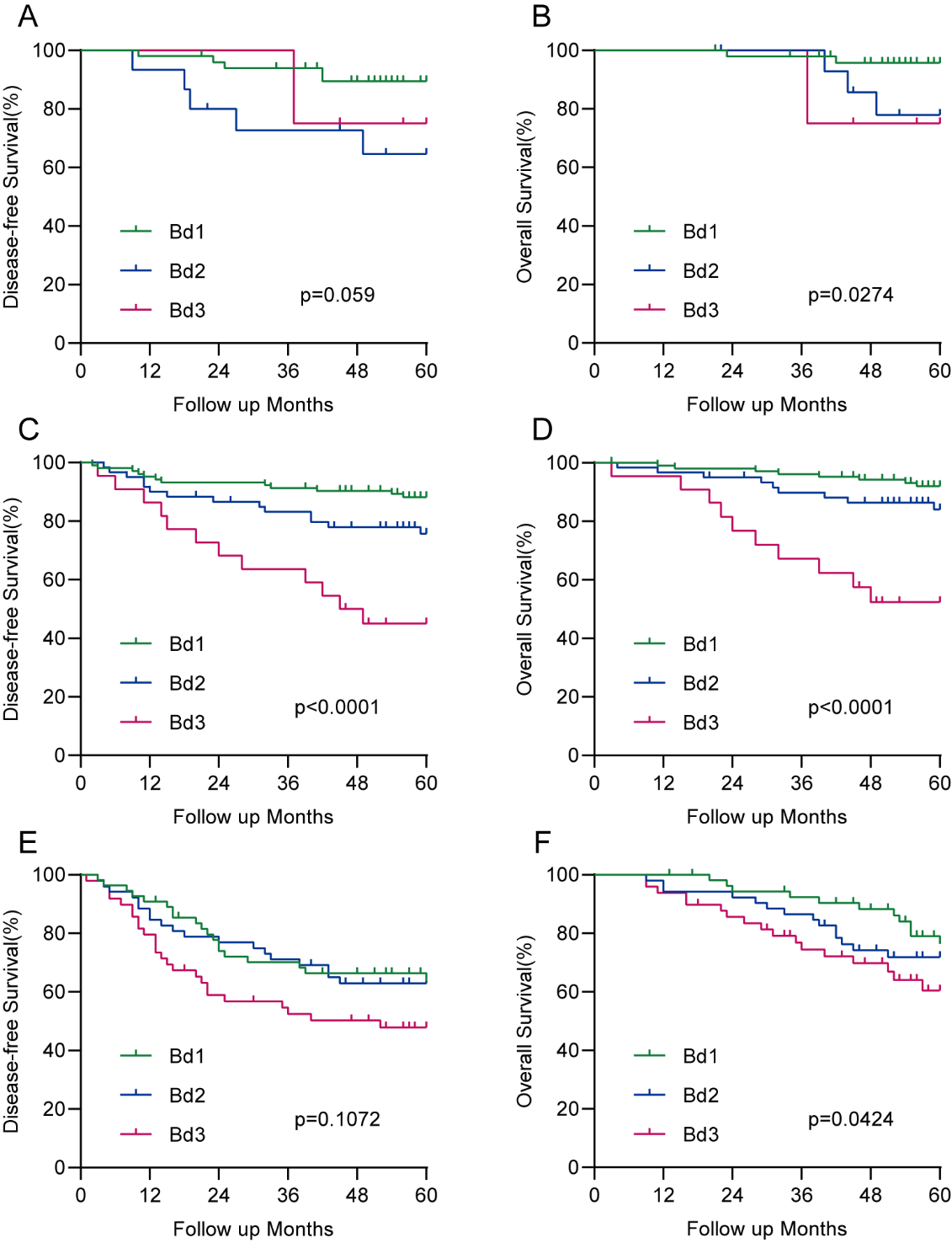
**

**Figure S4.** Survival outcomes according to TB stratified by TNM stage

Disease-free survival (A), overall survival (B) according to tumor budding in patients with stage I colorectal cancer.

Disease-free survival (C), overall survival (D) according to tumor budding in patients with stage II colorectal cancer.

Disease-free survival (C), overall survival (D) according to tumor budding in patients with stage III colorectal cancer.

**
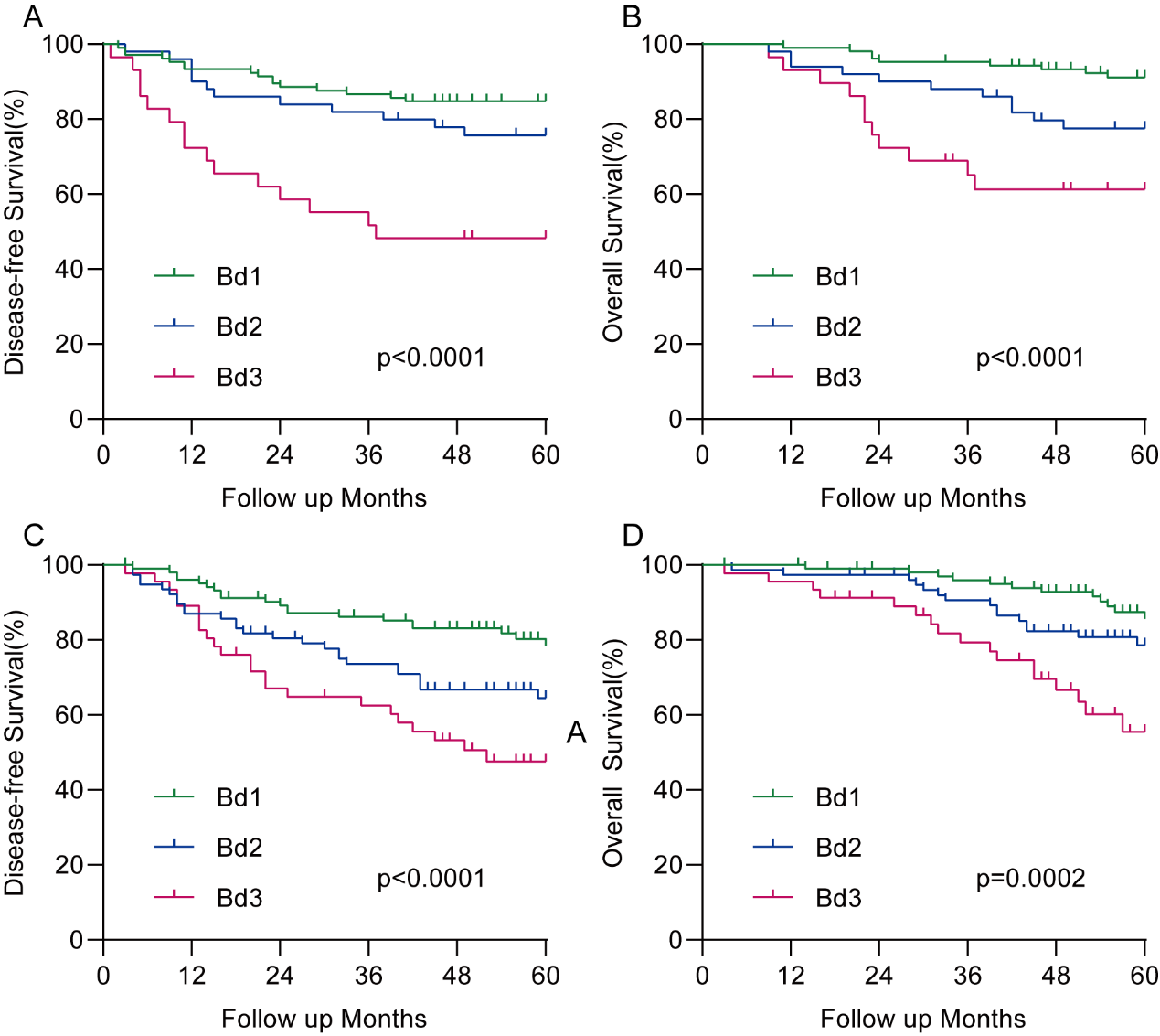
**

**Figure S5.** Survival outcomes according to TB stratified by tumor location

Disease-free survival (A), overall survival (B) according to tumor budding in patients with colon cancer.

Disease-free survival (C), overall survival (D) according to tumor budding in patients with rectal cancer.

**
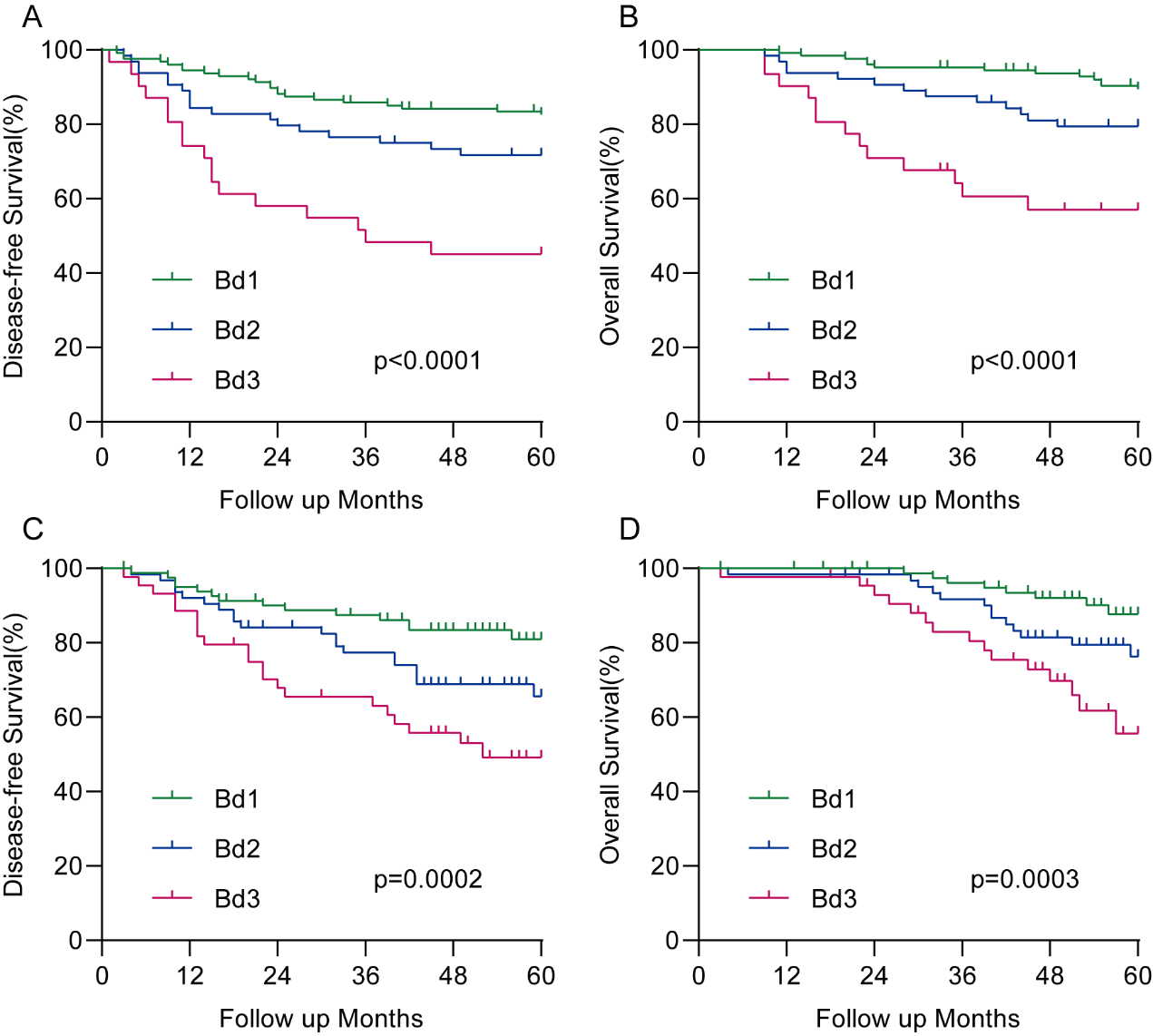
**

**Figure S6.** Survival outcomes according to TB stratified by MMR status

Disease-free survival (A), overall survival (B) according to tumor budding in patients with dMMR status.

Disease-free survival (C), overall survival (D) according to tumor budding in patients with pMMR status.

**
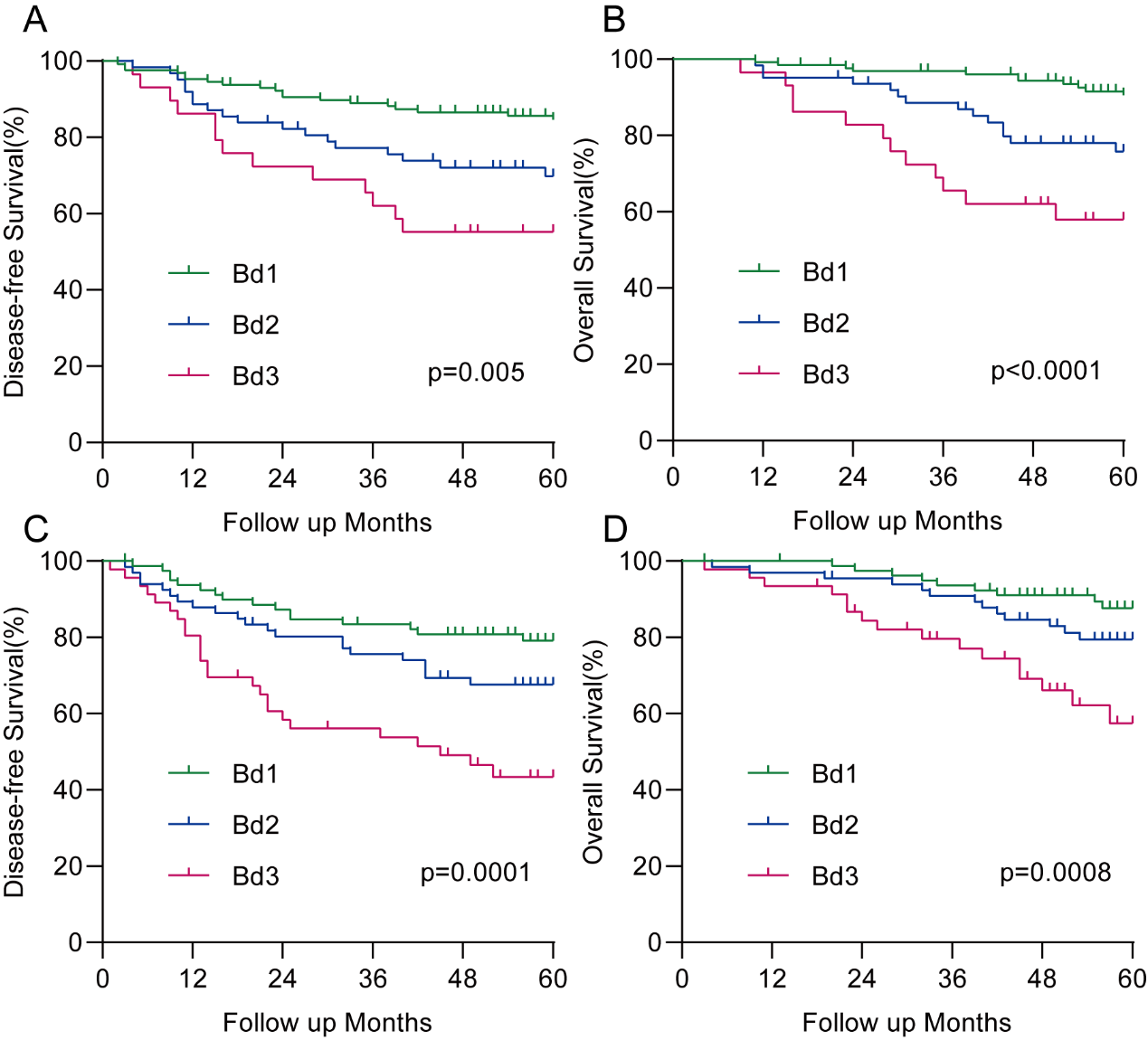
**

**Figure S7.** Survival outcomes according to TB stratified by LI

Disease-free survival (A), overall survival (B) according to tumor budding in patients with high LI.

Disease-free survival (C), overall survival (D) according to tumor budding in patients with low LI.


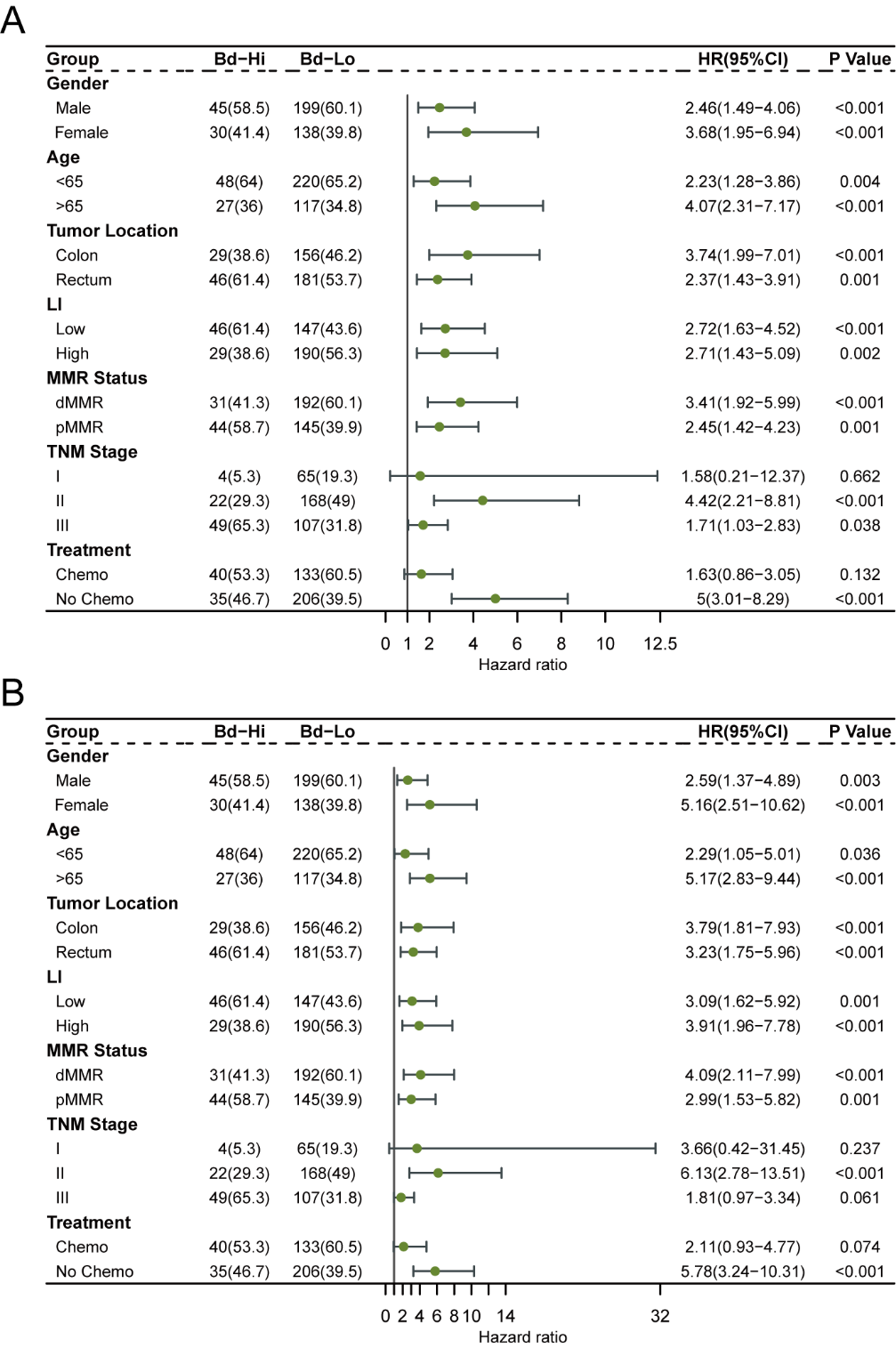


**Figure S8.** Subgroup analysis according to tumor budding grade 3 (Bd-Hi) vs tumor budding grade 2 and 1(Bd-Lo)

Subgroup analysis and forest plots for DFS (C) and OS (D) illustrating the hazard ratio according to tumor budding grade 3 (Bd-Hi) vs tumor budding grade 2 and 1(Bd-Lo).


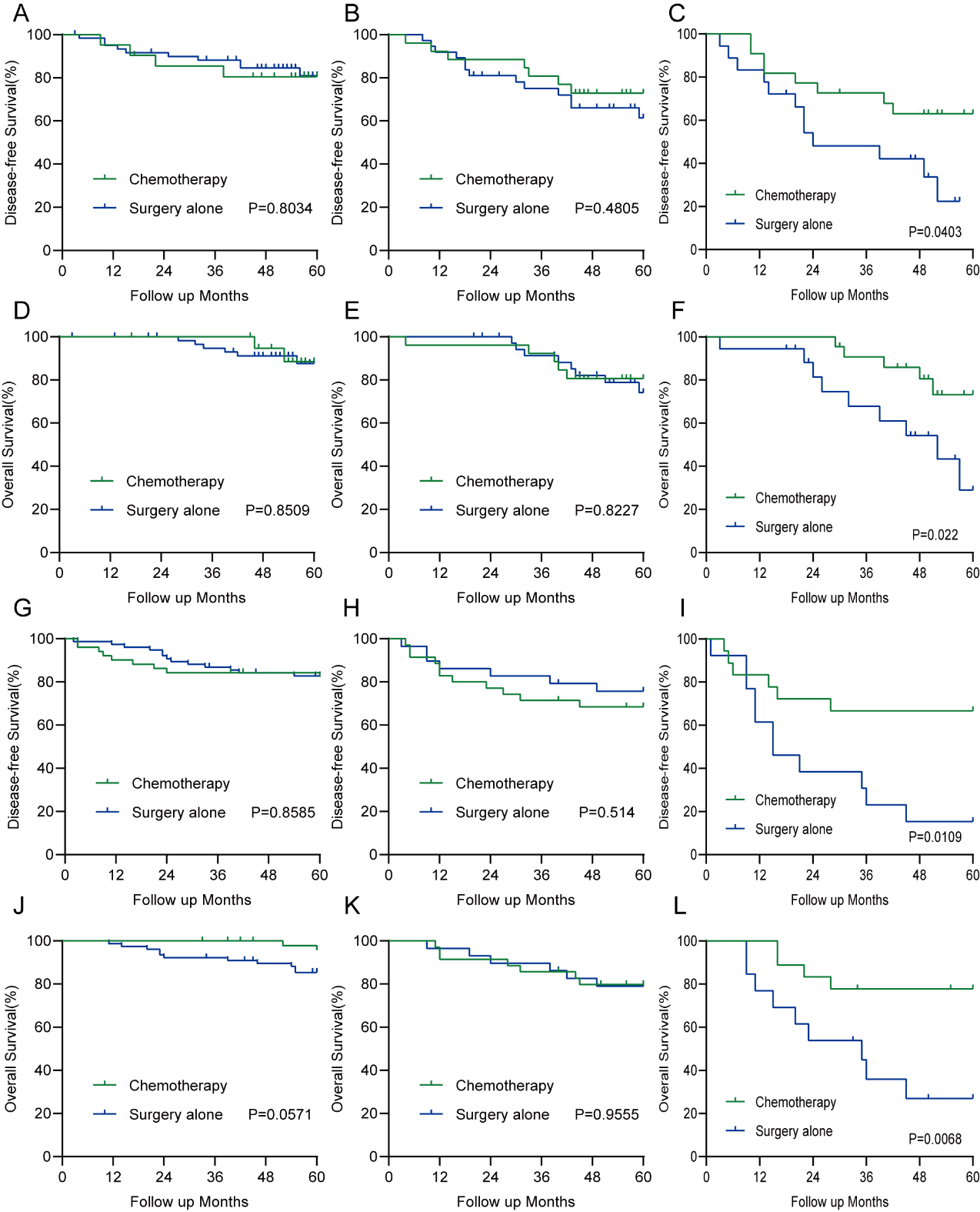


**Figure S9.** Effects of TB on chemotherapy stratified by MMR status

Disease-free survival according to treatment in Bd1 (A), Bd2 (B), Bd3 (C) groups in patients with pMMR status.

Overall survival according to treatment in Bd1 (D), Bd2 (E), Bd3 (F) groups in all patients with pMMR status.

Disease-free survival according to treatment in Bd1 (G), Bd2 (H), Bd3 (I) groups in patients with dMMR status.

Overall survival according to treatment in Bd1 (J), Bd2 (K), Bd3 (L) groups in all patients with dMMR status.

**
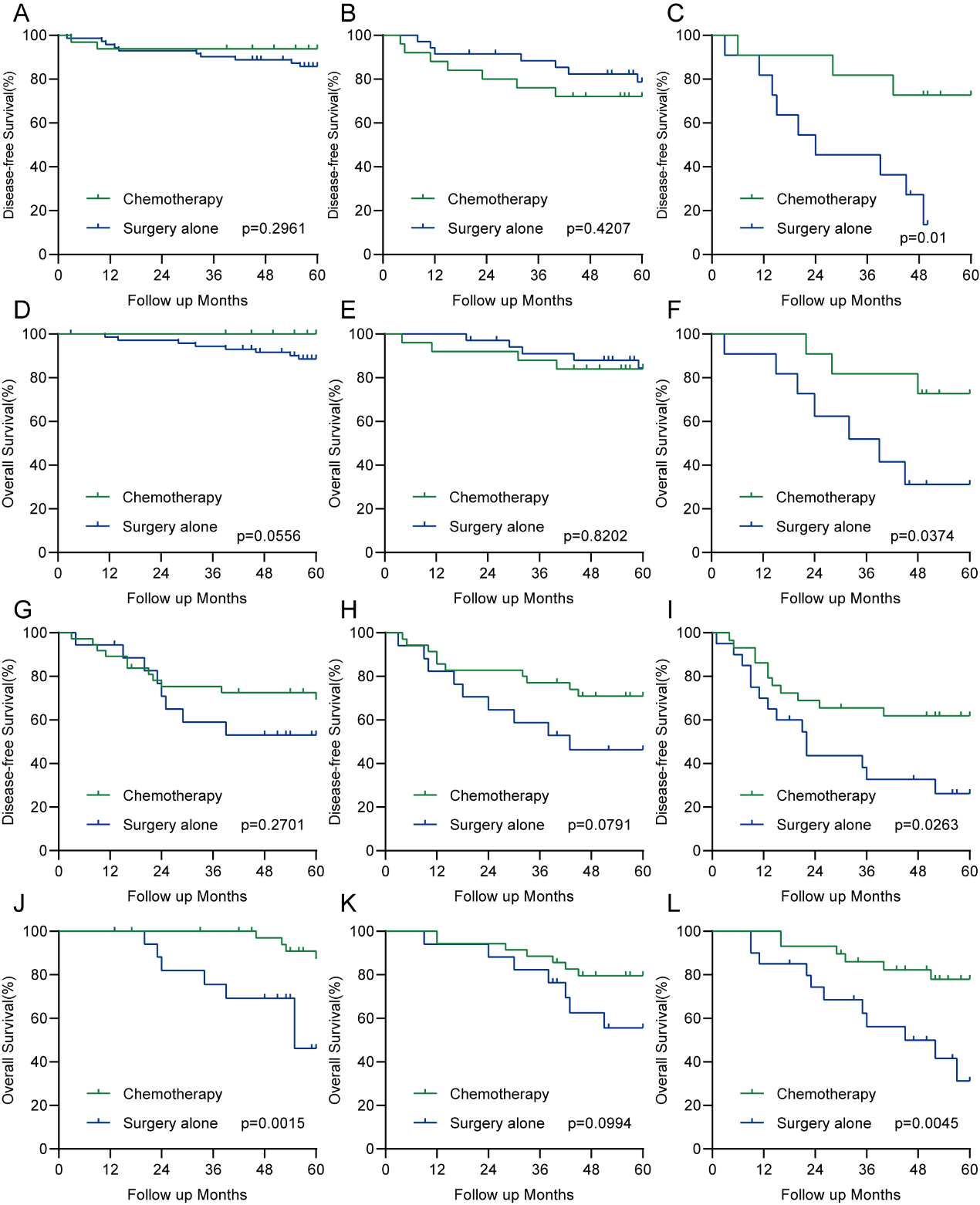
**

**Figure S10.** Effects of TB on chemotherapy stratified by TNM stage

Disease-free survival according to treatment in Bd1 (A), Bd2 (B), Bd3 (C) groups in patients with stage II colorectal cancer.

Overall survival according to treatment in Bd1 (D), Bd2 (E), Bd3 (F) groups in all patients with stage II colorectal cancer.

Disease-free survival according to treatment in Bd1 (G), Bd2 (H), Bd3 (I) groups in patients with stage III colorectal cancer.

Overall survival according to treatment in Bd1 (J), Bd2 (K), Bd3 (L) groups in all patients with stage III colorectal cancer.

**
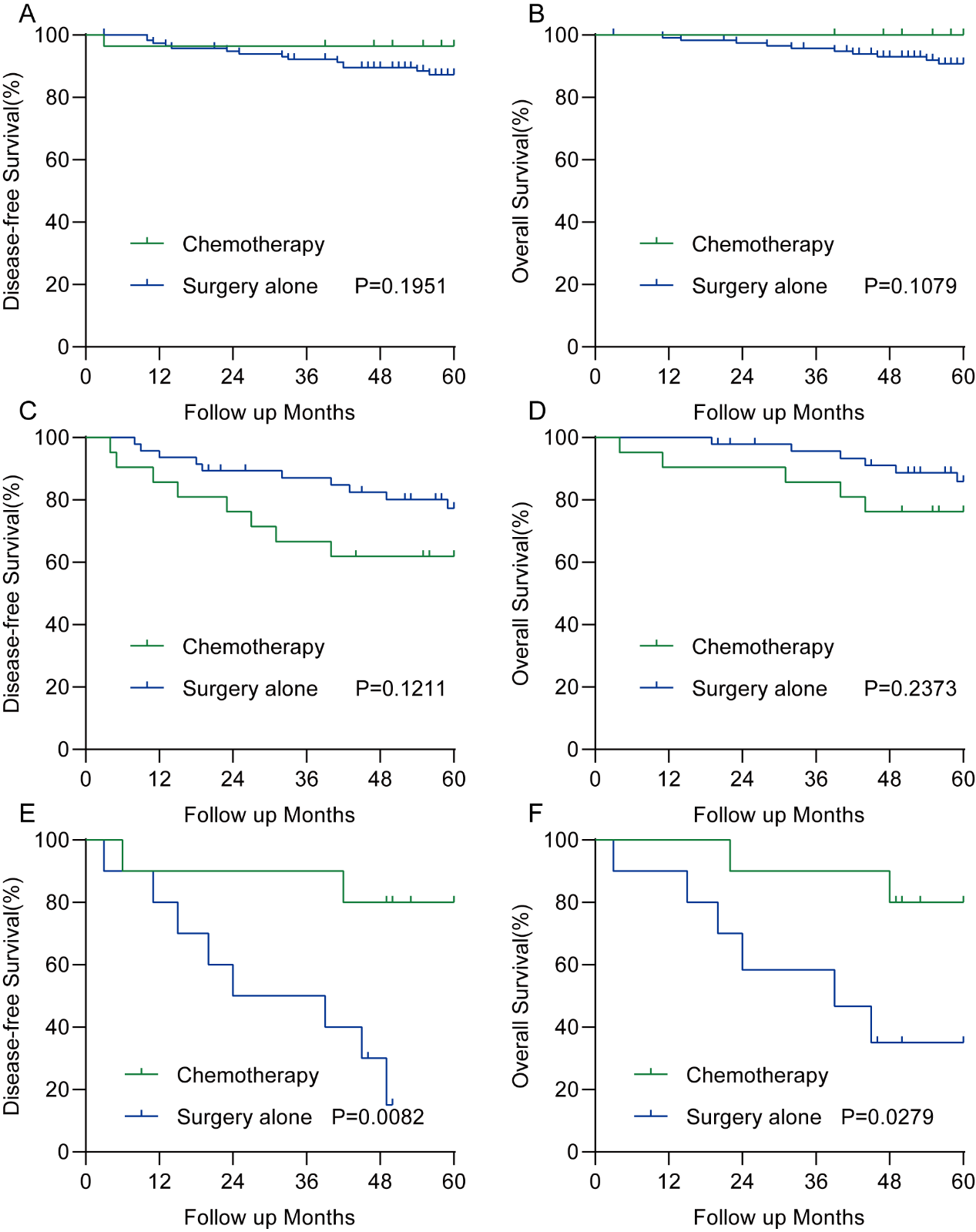
**

**Figure S11.** Effects of TB on chemotherapy in patients with T3N0 CRC

Disease-free survival according to treatment in Bd1 (A), Bd2 (B), Bd3 (C) groups in patients with T3 N0 stage colorectal cancer.

Overall survival according to treatment in Bd1 (D), Bd2 (E), Bd3 (F) groups in all patients with T3 N0 stage colorectal cancer.

**
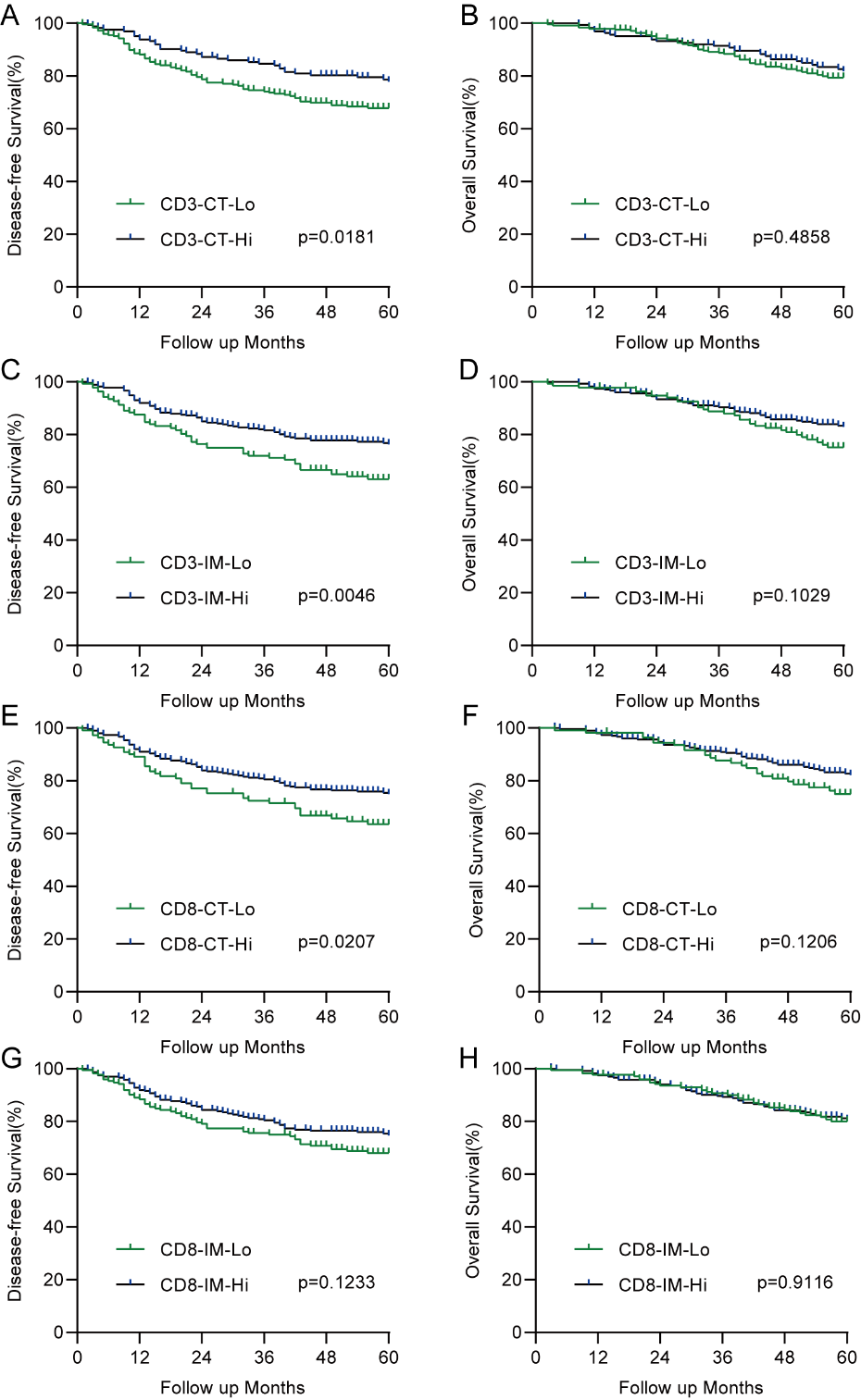
**

**Figure S12**. Disease-free survival (A), overall survival (B) according to CD3+ T cells in the center tumor (CT) in all patients

Disease-free survival (C), overall survival (D) according to CD3+ T cells in the invasive margin of tumor (IM) in all patients.

Disease-free survival (E), overall survival (F) according to CD8+ T cells in the center tumor (CT) in all patients.

Disease-free survival (E), overall survival (F) according to CD8+ T cells in the invasive margin of tumor (IM) in all patients.

**
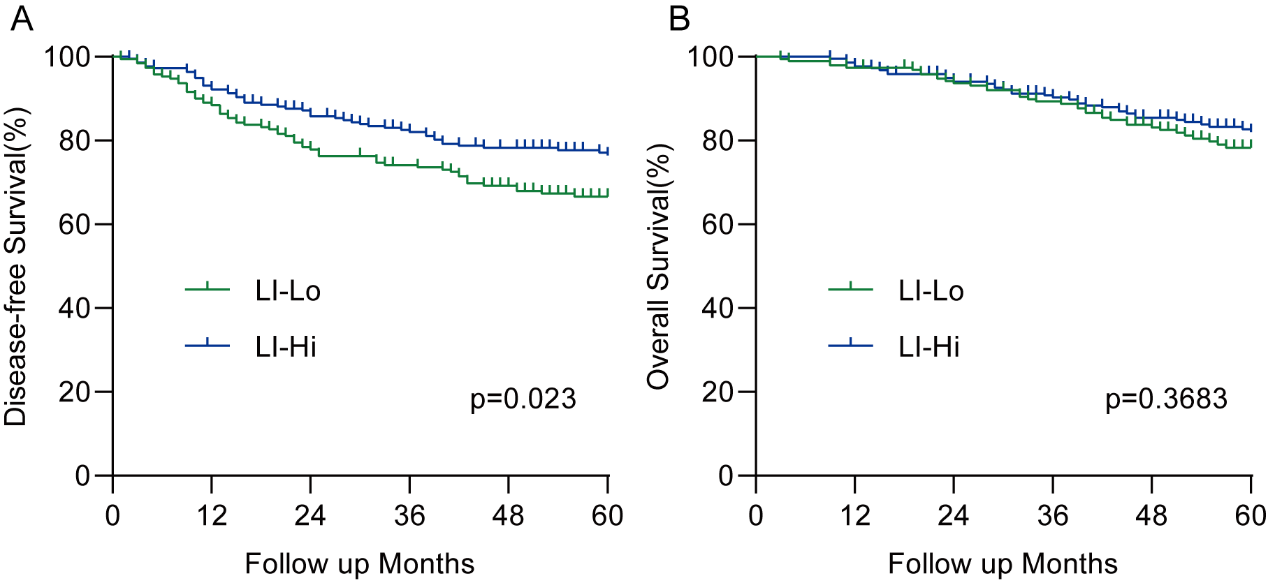
**

**Figure S13.** Survival outcomes according to lymphocytic infiltration (LI) in all patients

Disease-free survival (A), overall survival (B) according to lymphocytic infiltration (LI) in all patients.

**
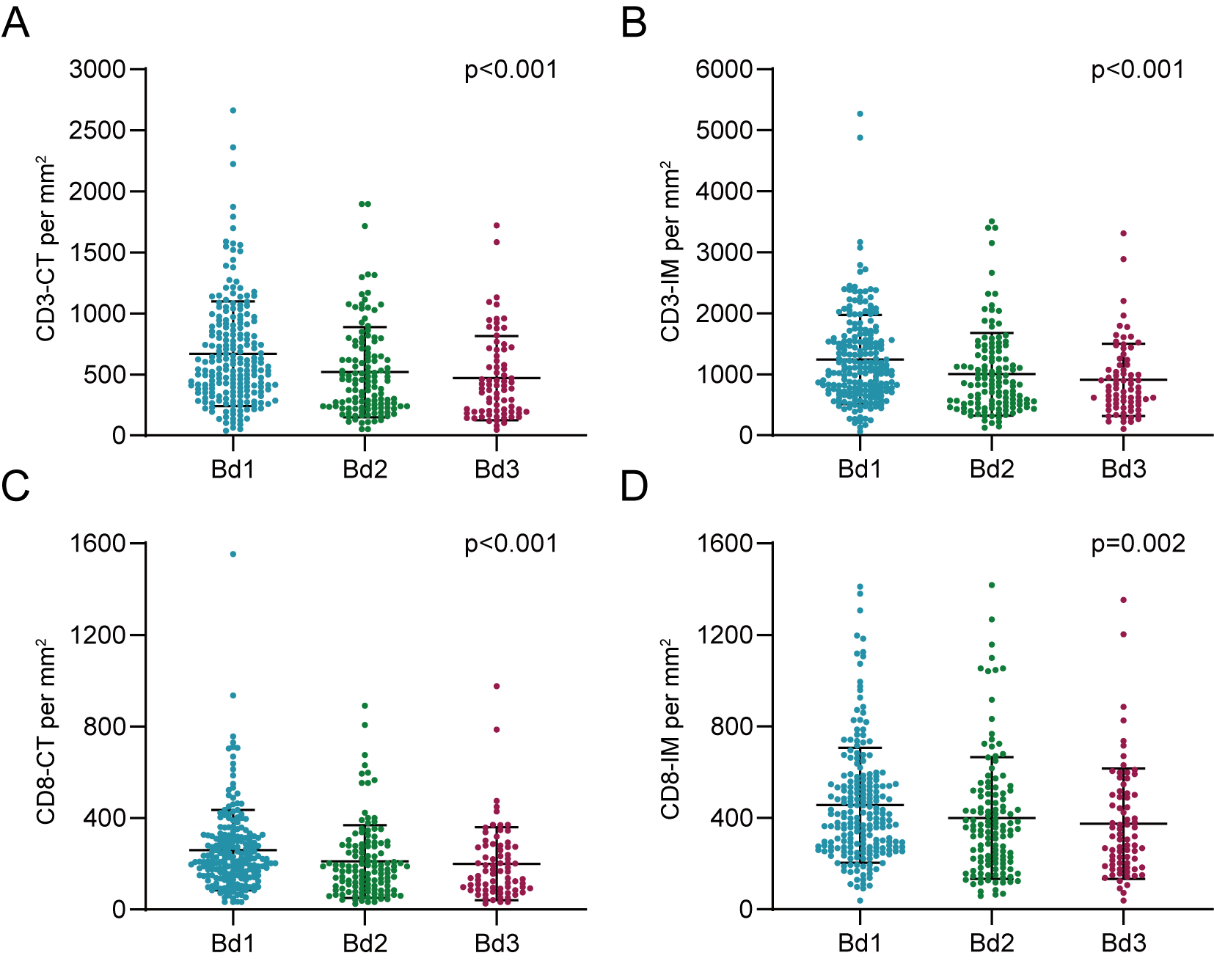
**

**Figure S14.** Association between TB and T cells

A, B, C, D, Scatter plot of number per mm^2^ of CD3+ and CD8+ cells in both the center of tumor (CT) and the invasive margin (IM) of the tumor stratified according to tumor budding grade with median and interquartile range.

**Table S1.** Optimal cutoff points for the four features were calculated based on disease-free survival

| Features | Cut-off value |
| --- | --- |
| CD3⁺ density (cells/mm²) in IM | 593 |
| CD3⁺ density (cells/mm²) in CT | 710 |
| CD8⁺ density (cells/mm²) in IM | 124 |
| CD8⁺ density (cells/mm²) in CT | 388 |

Abbreviations: IM: invasive margin; CT: center of tumor

**Table S2.** Prognosis predicted by TB

| **Disease-free Survival** | | | | | **Overall Survival** | | | |
| --- | --- | --- | --- | --- | --- | --- | --- | --- |
|  | Univariable HR (95% CI) | P value | Multivariable HR (95% CI) | P value | Univariable HR (95% CI) | P value | Multivariable HR (95% CI) | P value |
| pT stage |  |  |  |  |  |  |  |  |
| T1-T2 | 1 | **0.002** | 1 | 0.052 | 1 | 0.011 | 1 | 0.133 |
| T3-T4 | 2.82(1.47-5.41) |  | 1.93(0.99-3.77) |  | 2.95(1.29-6.79) |  | 1.99(0.85-4.69) |  |
| pN stage |  |  |  |  |  |  |  |  |
| N0 | 1 | **<0.001** | 1 | **0.02** | 1 | **<0.001** | 1 | 0.15 |
| N1-N2 | 2.81(1.91-4.09) |  | 1.72(1.09-2.73) |  | 2.65(1.67-4.21) |  | 1.51(0.86-2.65) |  |
| Tumor Deposits |  |  |  |  |  |  |  |  |
| Negative | 1 | **<0.001** | 1 | 0.202 | 1 | **<0.001** | 1 | 0.554 |
| Positive | 2.89(1.89-4.39) |  | 1.38(0.84-2.26) |  | 2.61(1.56-4.36) |  | 1.21(0.65-2.21) |  |
| Lymphovascular Invasion |  |  |  |  |  |  |  |  |
| Negative | 1 | **0.008** | 1 | 0.638 | 1 | **0.033** | 1 | 0.914 |
| Positive | 1.94(1.18-3.18) |  | 1.13(0.66-1.93) |  | 1.91(1.05-3.48) |  | 1.03(0.54-1.96) |  |
| Perineural Invasion |  |  |  |  |  |  |  |  |
| Negative | 1 | **<0.001** | 1 | **0.025** | 1 | **<0.001** | 1 | **0.018** |
| Positive | 2.81(1.89-4.33) |  | 1.71(1.06-2.73) |  | 3.13(1.87-5.23) |  | 1.94(1.11-3.39) |  |
| LI |  |  |  |  |  |  |  |  |
| Low | 1 | **0.024** | 1 | 0.088 | 1 | 0.36 | 1 | 0.907 |
| High | 0.65(0.44-0.94) |  | 0.71(0.48-1.05) |  | 0.81(0.51-1.27) |  | 0.97(0.61-1.55) |  |
| TBG |  |  |  |  |  |  |  |  |
| Bd1 | 1 |  | 1 |  | 1 |  | 1 |  |
| Bd2 | 1.89(1.21-2.98) | **<0.001** | 1.47(0.92-2.35) | 0.101 | 2.22(1.25-3.94) | **<0.001** | 1.85(1.03-3.34) | **0.039** |
| Bd3 | 3.79(2.41-5.99) | **<0.001** | 2.21(1.33-3.64) | **0.002** | 5.06(2.86-8.94) | **<0.001** | 3.26(1.73-6.13) | **<0.001** |

Abbreviations: pT stage: pathologic T stage; pN stage: pathologic N stage; LI: lymphocytic infiltration; TBG: tumor budding grade;

**Table S3.** Characteristics across patients with Bd 1, Bd2 and Bd3 between chemotherapy and surgery alone group

|  | Bd1 | | | | Bd2 | | | | Bd3 | | | |
| --- | --- | --- | --- | --- | --- | --- | --- | --- | --- | --- | --- | --- |
|  | Total | No-Chemo | Chemo | P value | Total | No-Chemo | Chemo | P value | Total | No-Chemo | Chemo | P value |
|  | 210 | 138 | 72 |  | 127 | 66 | 61 |  | 75 | 35 | 40 |  |
| Age in years, median (IQR) | 58(48,68) | 61(54,70) | 52(45,61) | **<0.001** | 58(49,68) | 64(53,72) | 55(45,64) | **<0.001** | 60(44,68) | 67(60,76) | 45(40,60) | **<0.001** |
| Sex, n (%) |  |  |  |  |  |  |  |  |  |  |  |  |
| Male | 124(59.1) | 82(59.4) | 42(58.333) | 0.879 | 75(59.1) | 39(59.1) | 36(59.1) | 0.993 | 45(60) | 19(54.2) | 26(65) | 0.345 |
| Female | 86(40.9) | 56(40.6) | 30(41.7) |  | 52(40.9) | 27(40.9) | 25(40.9) |  | 30(40) | 16(45.7) | 14(35) |  |
| Tumor Location, n (%) |  |  |  |  |  |  |  |  |  |  |  |  |
| Colon | 104(49.5) | 73(52.9) | 31(43.1) | 0.176 | 50(39.4) | 21(31.8) | 29(47.5) | 0.07 | 29(38.6) | 13(37.1) | 16(40) | 0.8 |
| Rectum | 106(50.5) | 65(47.1) | 41(56.9) |  | 77(60.6) | 45(68.2) | 32(52.4) |  | 46(61.3) | 22(62.8) | 24(60) |  |
| pT Stage, n (%) |  |  |  |  |  |  |  |  |  |  |  |  |
| T1-T2 | 53(25.2) | 45(32.6) | 8(11.1) | **<0.001** | 22(17.3) | 16(24.2) | 6(9.8) | **0.032** | 7(9.3) | 5(14.2) | 2(5) | 0.168 |
| T3-T4 | 157(74.8) | 93(67.4) | 64(88.9) |  | 105(82.7) | 50(75.8) | 55(90.2) |  | 68(90.6) | 30(85.7) | 38(95) |  |
| pN Stage, n (%) |  |  |  |  |  |  |  |  |  |  |  |  |
| N0 | 156(74.3) | 121(87.7) | 35(48.6) | **<0.001** | 71(55.9) | 47(71.2) | 24(39.3) | **<0.001** | 25(33.3) | 15(42.8) | 10(25) | 0.102 |
| N1-2 | 54(25.7) | 17(12.3) | 37(51.4) |  | 56(44.1) | 19(28.8) | 37(60.7) |  | 50(66.6) | 20(57.1) | 30(75) |  |
| Tumor Deposits, n (%) |  |  |  |  |  |  |  |  |  |  |  |  |
| Negative | 191(90.9) | 132(95.6) | 59(81.9) | **0.001** | 108(85.1) | 61(92.4) | 47(77.1) | **0.015** | 53(70) | 26(74.3) | 27(67.5) | 0.52 |
| Positive | 19(9.1) | 6(4.3) | 13(18.1) |  | 19(14.9) | 5(7.6) | 14(22.9) |  | 22(29) | 9(25.7) | 13(32.5) |  |
| Lymphovascular Invasion, n (%) |  |  |  |  |  |  |  |  |  |  |  |  |
| Negative | 196(93.3) | 132(95.6) | 64(88.8) | 0.062 | 115(90.5) | 60(90.9) | 55(90.2) | 0.886 | 55(73.3) | 27(77.1) | 28(70) | 0.485 |
| Positive | 14(6.6) | 6(4.3) | 8(11.1) |  | 12(9.4) | 6(9.1) | 6(9.8) |  | 20(26.6) | 8(22.8) | 12(30) |  |
| Perineural Invasion, n (%) |  |  |  |  |  |  |  |  |  |  |  |  |
| Negative | 192(91.4) | 128(92.8) | 64(88.8) | 0.342 | 113(88.9) | 60(90.9) | 53(86.9) | 0.469 | 58(77.3) | 29(82.8) | 29(72.5) | 0.285 |
| Positive | 18(8.6) | 10(7.2) | 8(11.1) |  | 14(11.1) | 6(9.1) | 8(13.1) |  | 17(22.6) | 6(17.1) | 11(27.5) |  |
| LI, n (%) |  |  |  |  |  |  |  |  |  |  |  |  |
| Low | 82(39.1) | 59(42.8) | 23(31.9) | 0.127 | 65(51.2) | 35(53.1) | 30(49.2) | 0.665 | 46(61.3) | 24(68.5) | 22(55) | 0.229 |
| High | 128(60.9) | 79(57.2) | 49(68.1) |  | 62(48.8) | 31(46.9) | 31(50.8) |  | 29(38.6) | 11(31.4) | 18(45) |  |
| MMR status, n (%) |  |  |  |  |  |  |  |  |  |  |  |  |
| dMMR | 82(39.1) | 61(44.2) | 21(29.2) | **0.034** | 63(49.6) | 37(56.1) | 26(42.6) | 0.13 | 44(58.6) | 22(62.8) | 22(55) | 0.491 |
| pMMR | 128(60.9) | 77(55.8) | 51(70.8) |  | 64(50.4) | 29(43.9) | 35(57.4) |  | 31(41.3) | 13(37.1) | 18(45) |  |

Abbreviations: pT stage: pathologic T stage; pN stage: pathologic N stage; MMR: mismatch repair; dMMR: mismatch repair-deficient; pMMR: mismatch repair-proficient LI: lymphocytic infiltration;
